# Genome sequence of an indole-3-acetic acid and siderophores producing *Pseudomonas monsensis* SARCC-3054 with the potential as a microbial inoculant for enhanced crop production

**DOI:** 10.1128/MRA.00479-23

**Published:** 2023-08-01

**Authors:** Ahmed Idris Hassen, Langutani Sanger Khambani, Rian Pierneef

**Affiliations:** 1 ARC-Plant Health and Protection, Pretoria, South Africa; 2 Department of Plant and Soil Sciences, Faculty of Science, Engineering and Agriculture, University of Venda, Thohoyandou, Limpopo, South Africa; 3 Department of Biochemistry, Genetics and Microbiology, University of Pretoria, Pretoria, South Africa; 4 Centre for Bioinformatics and Computational Biology, University of Pretoria, Pretoria, South Africa; 5 SARChI Chair: Marine Microbiomics microbiome@UP, Department of Biochemistry, Genetics and Microbiology, University of Pretoria (UP), Hatfield, Pretoria, South Africa; University of Arizona, Tucson, Arizona, USA

**Keywords:** *Pseudomonas monsensis*, genome, contigs, indole-3-acetic-acid, annotation, sequence reads, siderophores

## Abstract

The genome of *Pseudomonas monsensis* strain SARCC-3054 was sequenced after being confirmed as a potential plant growth-promoting rhizobacteria in both *in vitro* and *in vivo* assays. The 6.3 MB genome has a GC content of 60.2% and is divided into 59 contigs that contain several plant beneficial genes and proteins.

## ANNOUNCEMENT

*Pseudomonas monsensis* SARCC-3054 is a Gram-negative, motile, aerobic bacterium isolated from the rhizosphere soil of pristine grassland at the Nylsvlei Nature Reserve (S24^o^39*'*50.0 E 28^o^39*'*54.4) in South Africa. The bacterium was isolated using the method described in reference (1) and was tested *in vitro*, along with other isolates, to detect the presence of certain beneficial plant growth-promoting traits and *in vivo* for its plant growth promotion in selected crops (2). Two important plant growth-promoting rhizobacteria (PGPR) traits exhibited by this bacterium in the *in vitro* test include the production of indole-3-acetic acid (IAA) and siderophores. The results of the assays showed that the bacterial strain Lb-fp1/3-1b, now identified as *Pseudomonas monsensis* SARCC-3054 in this study, resulted in the highest amount of IAA production (97.20 µg/m) and is also one of the strains that produced siderophores with a 10-mm yellow halo zone on Chrome Azurol Agar (2). Based on the results, this strain could be grouped under PGPR and has the potential to be developed as a microbial inoculant to promote growth and yield of selected crops such as maize (*Zea mays* L.). In order to have a better understanding of these useful traits as well as other metabolic pathways *of Pseudomonas monsensis* strain SARCC-3054, we sequenced and analyzed the genome. The bacterium was initially isolated using a 100 µL aliquot of serially diluted rhizosphere soil suspension plated on a solid Luria Bertani (LB) agar medium. After incubation at 28°C for 24 h, a single pure colony of the bacterium was grown in LB broth for 24 h on a rotary shaker at 28°C. One milliliter of the culture suspension was used to extract total DNA using the Wizard genomic DNA purification kit and protocol (Promega, Madison, WI, USA).

For the preparation of paired-end libraries, the MGIEasy DNA Library Prep Kit was used (MGI Tech Co., Ltd, China). The end repair and A-tailing mixture was prepared using 2 ng/µL of the DNA sample and a total reaction mix of 10 µL. The PCR was run with a reaction condition of 37°C, 10 min; 65°C, 15 min; and 4°C on hold with the heated lid on. After adapter ligation and cleanup of the ligated DNA, PCR amplification and cleanup of the PCR product were performed. The final product was sequenced on an MGI DNBSEQ-G400 sequencing platform at the Agricultural Research Council’s Biotechnology Platform, Onderstepoort, South Africa. Adapter sequences and low-quality bases were trimmed using Trimmomatic v.0.36, and the overlapping pairs were merged on KBase (3). This resulted in 6,911,350 raw reads and 1,031,431,000 bases with a mean read length of 150 bp, which were used to create a *de novo* assembly with SPAdes v.3.15.3 (4) on KBase. The assembly had a total length of 6,287,674 bp with a GC content of 60.2%. The genome is divided into 59 contigs with *N*_50_ and *L*_50_ values of 210,587 and 8, respectively. The length distribution of the contigs was as follows: 20 had a size of >100,000 bp and 39 were between 100 and 10,000 bp.

**Fig 1:**
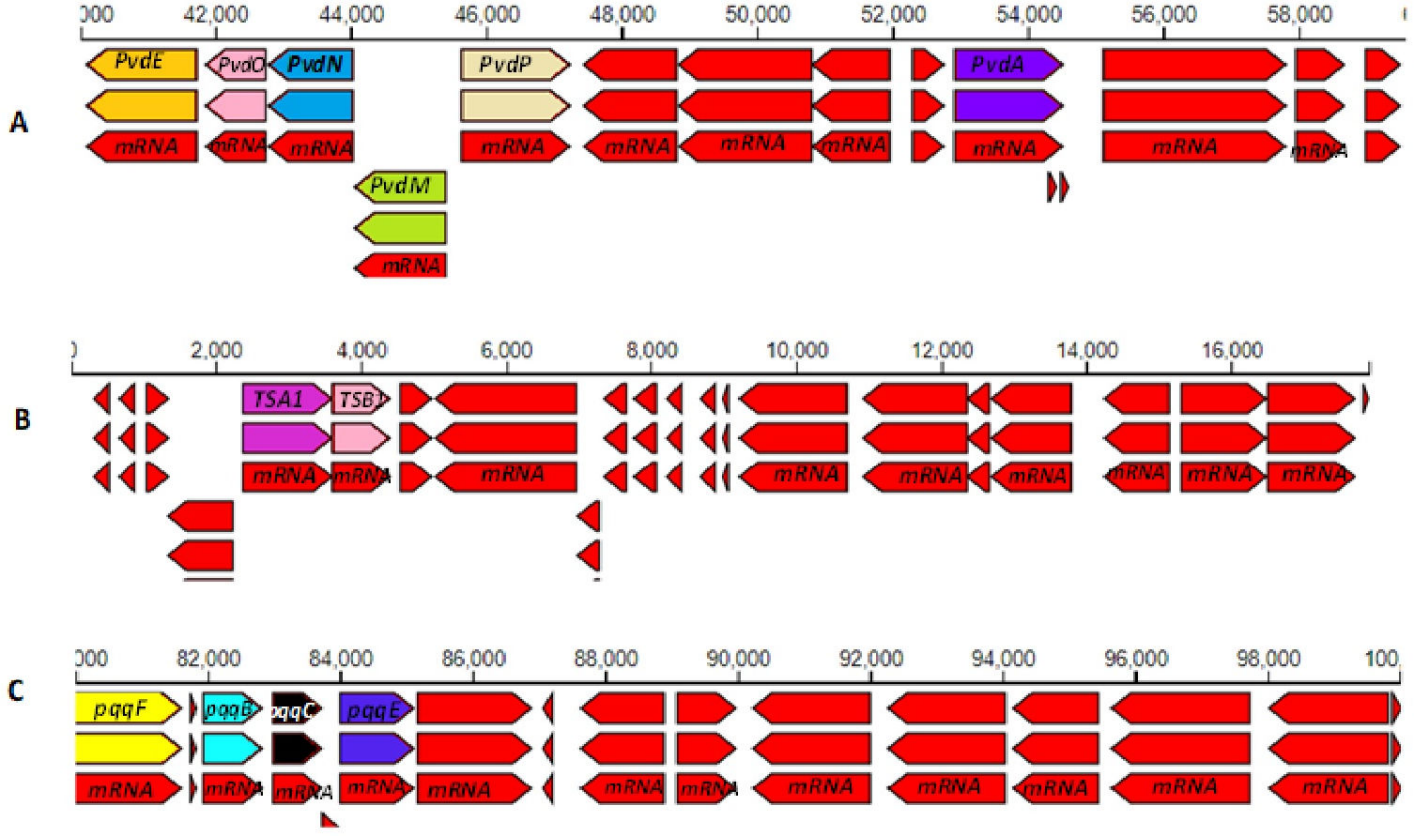
Chromosomal regions of annotated genes for selected PGPR traits of *Pseudomonas monsensis* SARCC-3054 generated on KBase. (A) Genes coding for the biosynthesis of pyoverdine (Pvd) siderophores *PvdE, PvdO, PvdN,* and *PvdM* (−) strands and *PvdP* and *PvdA* (+) strands. (B) Genes coding for enzymes involved in the biosynthesis of auxin, tryptophan synthase alpha (*TSA1*), and beta (*TSB1*) chain. (C) Genes coding for pyrroloquinoline quinone (PQQ) synthesis *pqqF, pqqB*, *pqqC,* and *pqqE* are all located on the (+) strand and serve as essential co-factors for the bacterial dehydrogenase involved in phosphate solubilization. All the remaining genes on the contigs with no labels and colored in red belong to other hypothetical proteins or coding genes.

Genome annotation was performed using the NCBI Prokaryotic Genome Annotation Pipeline (PGAP v.6.2) (5, 6) as well as the Rapid Annotation Using Subsystem Technology (RAST v.2) (7). The PGAP annotation is a publicly available annotation, while the RAST annotation was used for local investigation of pathways. In order to elucidate the features of various protein coding genes according to the Cluster of Orthologous Group (COG), Pfam, and TIGRfam assignments, the genome was further analyzed using the Integrated Microbial Genomes and Microbiomes pipeline of the *Joint Genome Institute* portal (8). Default parameters were used for all software unless otherwise specified. Table 1 and Fig. 1 summarize the statistics of the major genes and selected annotated genes of *Pseudomonas monsensis* SARCC-3054 involved with beneficial plant growth promotion, respectively. The strain was identified to species level as *Pseudomonas monsensis* using pairwise comparisons of the *dDDH* on the Type Strain Genome Server (9).

**TABLE 1:** Statistics of the major protein coding and other genes of *Pseudomonas monsensis* SARCC-3054

Genes total number	5,602	100%
Protein coding genes	5,465	97.5%
Regulatory and miscellaneous features	81	1.44%
RNA genes	66	1.17%
rRNA genes	5	0.09%
5S rRNA	1	0.02%
16S rRNA	2	0.03%
23S rRNA	2	0.03%
tRNA genes	57	1.02%
Other RNA genes	4	0.07%
Protein coding genes with function prediction	5,465	97.5%
Protein coding genes without function prediction	1023	18.26%
Protein coding genes with enzymes	1547	27.61%
Protein coding genes connected to KEGG^*a*^ pathways	1813	32.41%
Protein coding genes not connected to KEGG pathways	3755	67.10%
Protein coding genes connected to KEGG Orthology	3168	56.60%
Protein coding genes with Cluster of Orthologous Groups	4517	80.63%
with Pfam^*b*^	4768	85.11%
with TIGRfam^*c*^	1734	30.95%
with CATH FunFam^*d*^	3758	67.08%
Protein coding genes coding signal peptides	679	12.12%
Protein coding genes coding transmembrane proteins	1296	23.13%
COG clusters	2,211	39.46%
Pfam clusters	2,742	48.94%
TIGRfam clusters	1,320	23.56%

^*a*^ Kyoto Encyclopedia of Genes and Genomes, network of gene products including functional RNAs.

^*b*^ Protein families used in analyzing novel genomes or other functional regions.

^*c*^ A protein family resource for the functional identification of proteins.

^*d*^ A protein family used to predict functional sites in proteins and provide clues about putative function in novel protein sequences.

## Data Availability

The data have been deposited at DDBJ/ENA/GenBank under accession number JANIGP000000000. The version described in this paper is version JANIGP010000000. The BioProject accession number is PRJNA863286, and the SRA number is SRR21410270.
